# Crosstalk between vault RNAs and innate immunity

**DOI:** 10.1007/s11033-024-09305-y

**Published:** 2024-03-05

**Authors:** Rodolfo Gamaliel Avila-Bonilla, Juan Pablo Martínez-Montero

**Affiliations:** 1 Centro de Investigación y de Estudios Avanzados del Instituto Politécnico Nacional, Departamento de Genética y Biología Molecular, Av. IPN 2508, 07360 Mexico City, Mexico; 2 Universidad Autónoma del Estado de México (UAEMex), Toluca, Estado de México Mexico

**Keywords:** Vault RNAs, Innate immunity, RIG-I and PKR

## Abstract

**Purpose:**

Vault (vt) RNAs are noncoding (nc) RNAs transcribed by RNA polymerase III (RNA Pol III) with 5ʹ-triphosphate (5ʹ-PPP) termini that play significant roles and are recognized by innate immune sensors, including retinoic acid-inducible protein 1 (RIG-I). In addition, vtRNAs adopt secondary structures that can be targets of interferon-inducible protein kinase R (PKR) and the oligoadenylate synthetase (OAS)/RNase L system, both of which are important for activating antiviral defenses. However, changes in the expression of vtRNAs have been associated with pathological processes that activate proinflammatory pathways, which influence cellular events such as differentiation, aging, autophagy, apoptosis, and drug resistance in cancer cells.

**Results:**

In this review, we summarized the biology of vtRNAs and focused on their interactions with the innate immune system. These findings provide insights into the diverse roles of vtRNAs and their correlation with various cellular processes to improve our understanding of their biological functions.

## Introduction

In mammals, the innate immune system's primary defense against viral infections relies on type I interferon (IFN), a potent cytokine that directly interferes with various stages of the viral life cycle. This defense system depends on the ability of the immune system to differentiate between self- and nonself-nucleic acids [1]. To make this distinction, cells have developed a plethora of pattern recognition receptors (PRRs), including Toll-like receptors (TLRs) and RIG-I-like receptors (RLRs), which are capable of identifying specific pathogen-associated molecular patterns (PAMPs), such as viral nucleic acids [2]. Once a PAMP is identified, type I IFN is secreted and binds to the IFN receptor (IFNAR1/2). This binding initiates the JAK/STAT (Janus kinase-signal transducer and activator of transcription) signaling pathway, which promotes the transcription of more than 300 interferon-stimulated genes (ISGs), which have diverse functions and contribute to an efficient antiviral response [3–6].

RLRs, which include retinoic acid-inducible protein 1 (RIG-I), melanoma differentiation-associated gene 5 (MDA5), and laboratory of genetics and physiology 2 (LGP2), play crucial roles in recognizing the molecular and structural features of cytoplasmic virus-derived double-stranded (ds) RNA structures [7–9]. RIG-I preferentially binds short dsRNAs (< 300 bp) with uncapped blunt ends carrying a 5ʹ-PPP, 5ʹ-diphosphate (5ʹ-PP) groups, and partially methylated 5ʹ-terminal nucleotides, including m7G Cap0 dsRNAs, as well as metabolite-capped dsRNAs such as 5ʹ-CoA-RNAs, 5ʹ-NAD+-RNAs, and 5ʹ-FAD-RNAs [10–17]. Conversely, MDA5 serves as a cytosolic RNA sensor that specifically interacts with long dsRNA molecules (> 1 Kbp), forming fibers along the dsRNA in collaboration with LGP2 [18, 19]. RIG-I and MDA5 can trigger the nuclear translocation of transcription factors such as interferon regulatory factor 3 (IRF3), IRF7, and nuclear factor-kB (NF-kB). These transcription factors cooperate to initiate the expression of type I IFN and proinflammatory cytokines, such as tumor necrosis factor-α (TNF-α), interleukin 6 (IL-6), and IL-8 [12, 20–23].

In addition to type I IFN production, the presence of cytoplasmic-viral dsRNA can trigger other pathways that are important for antiviral defense. These processes inhibit host RNA translation and initiate RNA degradation [24, 25]. For instance, the recognition of dsRNA results in the activation of interferon-inducible protein kinase R (PKR), which phosphorylates eukaryotic initiation factor 2 α (eIF2α) to block cap-dependent mRNA translation. This activation also triggers NF-kB, promoting the expression of proinflammatory cytokines such as interleukin 1 (IL-1) and TNFα [26–29]. Similarly, the IFN-stimulated oligoadenylate synthetase (OAS)/RNase L system, which is triggered by cytosolic virus-derived dsRNA, leads to the generation of 2′–5′ oligoadenylates, thereby promoting the dimerization and activation of RNase L, an endonuclease responsible for the widespread degradation of cytoplasmic mRNA [30–33].

While the innate immune system has evolved to recognize viral RNA, it is crucial to realize that endogenous RNAs can also serve as substrates for classic nucleic acid sensors. This recognition has the potential to initiate an innate antiviral response, which may contribute to the development of several pathologies, including autoimmune diseases, cancer, and premature aging [34–36]. In mammals, a significant number of noncoding RNAs (ncRNAs) transcribed by RNA polymerase III (RNA Pol III) bear 5ʹ-triphosphate (5ʹ-PPP) termini, suggesting that these RNAs can trigger viral nucleic acid sensors [37]. This group of RNA Pol III-transcribed ncRNAs includes transfer RNAs (tRNAs), 7SL RNAs, 7SK RNAs, 5S rRNA, U6 small nuclear RNAs (snRNAs), YRNAs, Alu RNAs, and vault (vt) RNAs [38–43]. Furthermore, these ncRNAs have been linked to pathological processes associated with systemic inflammation, which is often driven by the abnormal production of interferon type I (IFN-I) [44–47].

vtRNAs have been associated with various diseases, including cancer, neurodegenerative disorders, nephropathy, skin aging, autoimmune diseases, and diabetes. These conditions involve an immunological component, although the precise mechanisms of vtRNAs have not been determined [48–50]. While vtRNAs are primarily related to homeostasis and cellular proliferation, they are also transcribed by RNA polymerase III, suggesting their potential recognition by RNA sensors. This review aims to provide insights into vtRNA biology, interactions with innate immune system proteins, roles in antiviral responses and immunological diseases, and involvement in other cellular processes, including potential contributions to pathways such as NF-kB activation.

## Vault RNA biogenesis

Vault (vt) RNAs are small, evolutionarily conserved noncoding RNAs [51]. These factors were initially identified as integral components of ribonucleoproteins known as vault particles [52–57]. These particles consist of three proteins (major vault protein (MVP), vault poly (ADP-ribose) polymerase (VPARP), and telomerase-associated protein 1 (TEP1)) that are often associated with one or more vtRNA molecules [58–60]. Notably, approximately 95% of vtRNA molecules are located in the cytoplasm as free molecules with sizes ranging from 80 to 140 nucleotides (nt), indicating that vtRNAs can operate independently of MVP [54, 55].

**Fig. 1:**
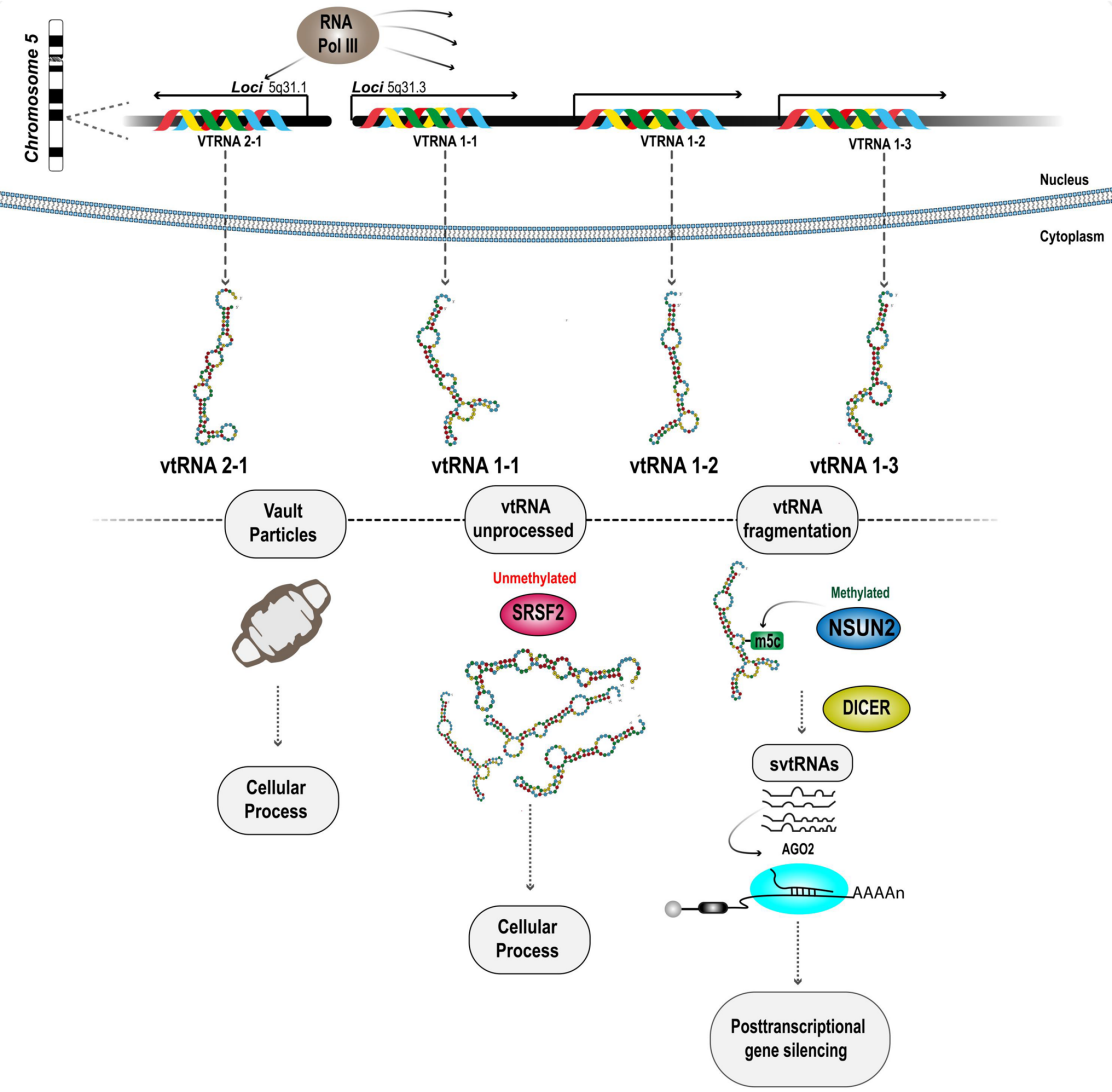
**The human vault RNAs biogenesis.** The four vtRNAs are encoded on two loci on chromosome 5. vtRNA 1-1, vtRNA 1-2, vtRNA 1-3 encode on locus 5q31.3, while vtRNA 2-1 is encoded on locus 5q31. All vtRNAs are expressed by RNA polymerase III [57–59]. vtRNAs can be associated with vault particles which are involved in different cellular processes [58, 64]. vtRNAs may remain unprocessed and protected from methylation by the SRSF2 protein. Full-length vtRNAs can participate in several cellular processes [73, 74]. Additionally, vtRNAs may undergo fragmentation through m5C methylation at position C69 by NSUN2 protein. This methylation facilitates the fragmentation into small vault RNAs (svtRNAs) by DICER. Some svtRNAs can be loaded into AGO2, regulating posttranscriptional gene silencing [65, 73]

In the human genome, vault RNAs are located on chromosome 5q31 and are distributed across two distinct loci [61–63]. The VAULT-1 locus houses three genes: vtRNA 1-1, vtRNA 1-2, and vtRNA 1-3 [55, 64]. Additionally, the VAULT-2 locus encodes vtRNA 2-1, which is also known as pre-miR-886 [64, 65]. The biogenesis of vtRNAs is poorly understood (Fig. 1). After being transcribed by RNA polymerase III, vtRNAs can mediate posttranscriptional gene silencing through a DICER-dependent processing mechanism. Notably, this mechanism is distinct from the canonical micro (mi)RNA pathway, and it operates independently of Drosha. This mechanism is exemplified by vtRNA 1-2 and vtRNA 2-1. After DICER-mediated processing, vtRNAs undergo fragmentation into small vault RNAs (svtRNAs), which are approximately 22–24 nt in length and can integrate into the Argonaute RISC Catalytic Component 2 (AGO2) protein. In particular, the integration of the small fragment derived from vtRNA1-2, which was designated svtRNA1-2, was observed. After being incorporated into AGO2, svtRNA1-2 plays a regulatory role, influencing the expression of genes associated with cell adhesion and cell membrane physiology [65].

It has been reported that vtRNA1-1 may not undergo cleavage by DICER [65, 66]. However, vtRNA1-1 may still serve as a specific substrate for this nuclease [67]. The processing of small fragments derived from vtRNA1-1 by DICER may lead to changes in gene expression. An illustrative example is svtRNA 1b, which has been shown to repress the expression of genes associated with drug resistance after being incorporated into AGO2 [68]. The differences in the processing of vtRNA1-1 may be attributed to the posttranscriptional modification of this noncoding RNA [69]. The 5-methylcytosine (m5C) modification has been suggested to be abundant in vtRNAs, and there is a notable occurrence of cytosine 69 (C69) methylation in vtRNA 1-1, and this modification is prevalent in human cells [70–72]. This methylation process is mediated by NOP2/Sun RNA Methyltransferase 2 (NSUN2) and plays a critical role in determining the processing of vtRNA 1-1 into svtRNA fragments that can regulate gene expression [73]. A clear example is the biogenesis of the svtRNA 4 fragment, which is generated as a product of C69 methylation of vtRNA 1-1 mediated by NSUN2. Without this protein, vtRNA 1-1 cannot produce fragments because it binds to the splicing factor protein arginine/serine-rich 2 (SFRS2), protecting it from methylation and cleavage [73, 74]. SRSF2 has been reported to bind to other RNA Pol III transcripts, such as 7SK. The formation of the 7SK riboprotein complex facilitates the association with promoter sites, thereby leading to the transcriptional activation of nascent RNAs [75]. Additionally, vtRNAs have been reported to exhibit nuclear localization, suggesting that unmethylated vtRNAs could have similar functions [65].

Moreover, not all svtRNAs undergo processing by AGO2, as exemplified by vtRNA 2-1. Despite being processed by DICER, fragments of vtRNA 2-1 are not loaded into the AGO2 complex [65]. A possible reason for this difference could be the absence of methylation, which is analogous to the mechanism of miRNAs. m5C methylation of miRNAs facilitates the loading of these small RNAs into AGO, enabling them to mediate posttranscriptional gene silencing [76]. Finally, m5C methylation has been shown to improve the stability and proper folding of noncoding RNAs (ncRNAs). This effect is observed in tRNAs, and the presence of these methylations throughout the molecule protects against degradation by nucleases and facilitates codon‒anticodon interactions [77, 78]. Moreover, vtRNAs are known to be susceptible to degradation by DIS3-like 3ʹ–5ʹ exoribonuclease 2 (DIS3L2), which plays a role in regulating their concentration and stability within the cell [79]. However, it is unknown whether the methylation of vtRNAs helps protect these molecules from degradation by ribonucleases. Additional information is required to determine these processing mechanisms.

## Vault RNAS and innate immunity

The interaction between innate immune components and vtRNAs has been the subject of study, but a complete understanding of this relationship is still lacking [59, 80]. Structural predictions suggest that vtRNAs 1-1, 1-2, and 1-3 adopt conformations that are recognized by the RIG-I receptor. This recognition is attributed to the fact that vtRNAs have 5ʹ-PPP termini, indicating that they trigger RIG-I receptors [81, 82]. Under normal physiological conditions, the 5ʹ-PPP termini of vtRNAs can be converted to 5ʹ-monophosphates (5ʹ-P) by the cellular triphosphatase dual-specificity phosphatase 11 (DUSP11), reducing their ability to trigger an innate immune response via RIG-I. However, during physiological stress events such as viral infections, DUSP11 can downregulate RIG-I expression, leading to an increase in the concentration of RNA species with 5ʹ-PPP groups at their terminal ends, thereby allowing recognition by RNA sensors and induction of the IFN response [46, 47, 82].

**Fig. 2:**
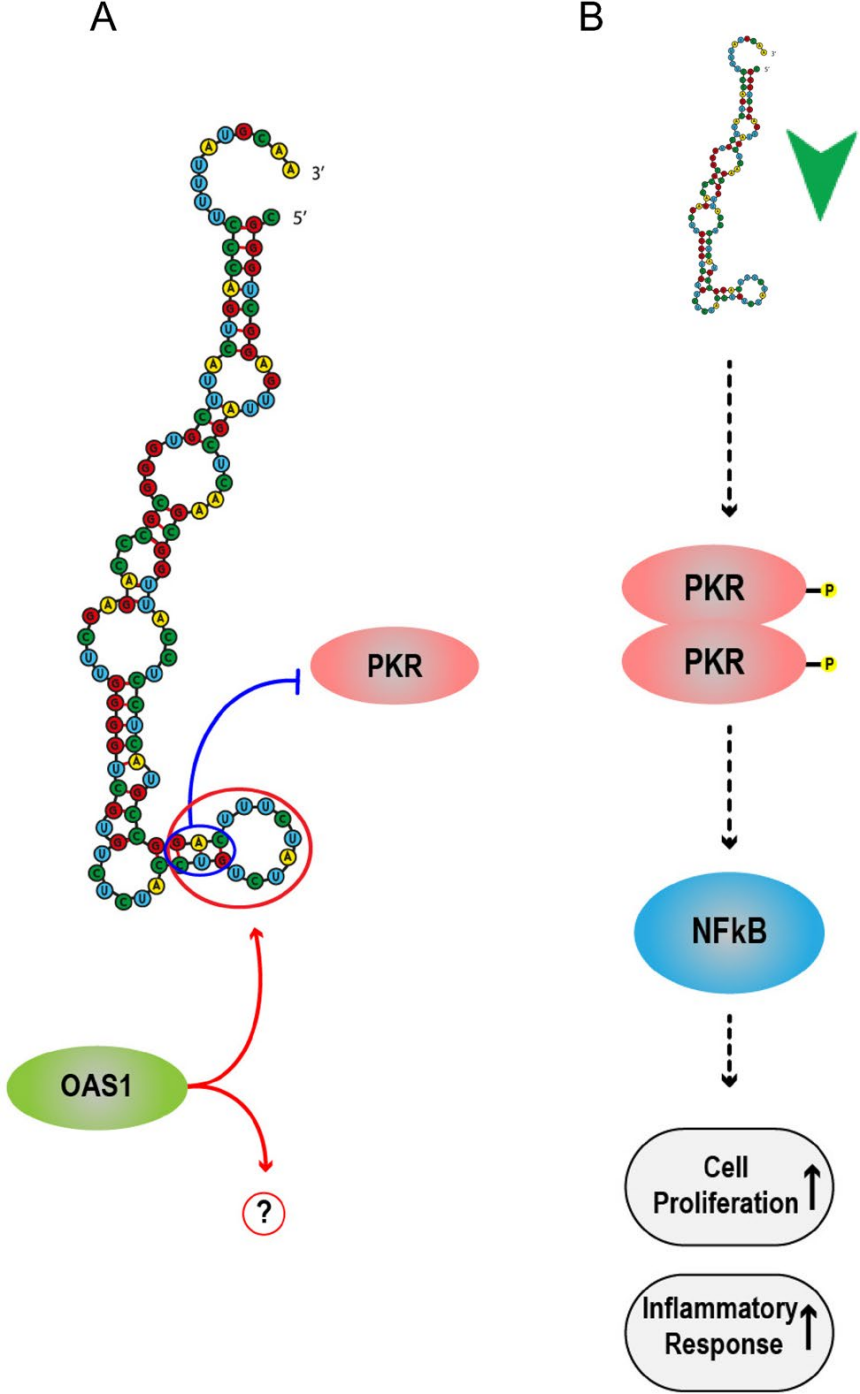
**Interactions of innate immune proteins with vtRNA 2-1.** PKR binds to vtRNA 2-1 at positions 40–42 nt, resulting in the unphosphorylated and inactivated PKR [84]. The essential nucleotides for PKR interactions are encircled in blue. OAS1 can bind to the apical stem-loop region (40–50 nt) of vtRNA 2-1. However, the details of this interaction remain unknown [89]. The critical nucleotides for OAS1 recognition are encircled in red. **B)** vtRNA 2-1 downregulation (green arrow) allows the activation of PKR through dimerization and phosphorylation and promotes the activation of NF-κB increasing proliferation and inflammatory response [84, 85]

Other types of proteins of the innate immune system can recognize vtRNAs; for example, the PKR protein, which is an interferon-stimulated gene (ISG), plays a crucial role in the host innate immune response. It serves as an RNA sensor that becomes activated in response to viral-derived dsRNAs and 5ʹ-PPP RNAs, triggering the NF-kB signaling pathway [83]. PKR can bind to vtRNA 2-1 at the apical stem, specifically at 40–42 nt, where the noncoding RNA adopts a double-stranded RNA structure (Fig. 2) [84, 85]. Upon binding to this site, PKR remains inactive (unphosphorylated). Depletion of vtRNA 2-1 activates PKR through dimerization and phosphorylation. This activation subsequently leads to an increase in the phosphorylation of IκB, followed by its degradation and the activation of NF-κB [84]. The activation of NF-κB plays multiple roles in regulating cellular processes related to proliferation, development, apoptosis, and immune and inflammatory responses [86]. During NF-κB activation mediated by the PKR-vtRNA interaction, vtRNA 2-1 has been reported to increase cell proliferation [85]. Similarly, vtRNA 1-1 is activated by the transcription of NF-κB, stimulating the proinflammatory pathway, which is mediated by members of the TNFR superfamily and can participate in the inflammatory response to viral infection [87, 88].

Calderon and Conn [89] suggested that OAS1 identifies the apical stem‒loop 40–50 nt of vtRNA 2-1, leading to activation of the RNase L pathway in A549 cells. This site of vtRNA 2-1 is crucial for recognition by proteins of the innate immune system and likely serves as an important site that regulates inflammatory responses (Fig. 2). The OAS/RNase L pathway, which is activated by IFN, serves as a robust antiviral mechanism designed to recognize and degrade viral and self single-stranded RNA (ssRNA) in host cells [30]. Interestingly, in cultures of peripheral blood mononuclear cells (PBMCs) from patients with multisystem inflammatory syndrome who have autosomal recessive deficiencies in the OAS/RNase L pathway and in THP-1 monocyte cells depleted of OAS/RNase L, stimulation with synthetic dsRNA exacerbates the production of the inflammatory cytokines IFN-λ1, CXCL10, and IL-6. The opposite effect was obtained when RNase L expression was restored [90]. Furthermore, in pancancer studies, OAS1 was reported to be highly expressed in a wide range of tumors [91]. The cytokines IFN-λ1, CXCL10, and IL-6 contribute to antiproliferative phenotypes [92–94]. The expression of OAS1 in tumors likely mediates the restriction of these cytokines, playing an important role in the tumor immune response. However, in the context of the interaction of vtRNA 2-1 with OAS1 and the regulation of these inflammatory cytokines, the underlying mechanism remains unknown. Therefore, a deeper understanding of this interaction could provide insights into the regulation of this noncoding RNA in the context of innate immunity.

In addition, the expression of vtRNAs has been associated with other inflammatory mechanisms. For instance, exposure of human keratinocytes to UV-B radiation can induce inflammation by upregulating cyclooxygenase 2 (COX2) and TNF-α and activating matrix metalloproteinase-9 (MMP9), potentially leading to premature skin aging [95, 96]. Notably, in UV-B-exposed keratinocytes lacking vtRNA 2-1, PKR phosphorylation is triggered, resulting in increased NF-κB activity and increased expression of the proinflammatory cytokines TNF-α, MMP-9, and COX-2, thus exacerbating inflammation and accelerating skin aging [97].

Moreover, in the case of IgAN (IgA nephropathy), which is the most common primary glomerulonephritis, researchers have detected hypermethylation in the DNA region encoding vtRNA 2-1. This hypermethylation reduces the expression of vtRNA 2-1 and suppresses CD4^+^ T-cell proliferation. This reduction may be associated with the binding of vtRNA 2-1 to PKR, which inhibits the activity of NF-κB, a crucial factor in cell proliferation [98, 99]. Decreased levels of vtRNA 2-1 also result in increased expression of transforming growth factor-β (TGF-β) and decreased expression of tripartite motif containing 27 (TRIM27) [98]. Both proteins are important for the pathogenesis of this disease. TGF-β is required to regulate the production of immunoglobulin A (IgA) by stimulating B cells to control T cells. This process involves the interaction of CD40 ligand (CD40L) and the T-cell receptor (TCR) [100, 101]. It is possible that the reduction in vtRNA 2-1 stimulates B cells to generate IgA, leading to kidney damage [98]. TRIM27 negatively regulates CD4+ T cells by polyubiquitinating phosphoinositide 3-kinase (PI3K), a protein that is essential for activating Ca^2+^ flux, which triggers CD4^+^ T-cell activation [102]. It is possible that the reduction in vtRNA 2-1 contributes to the activation of CD4+ T cells by inhibiting the activity of TRIM27 in patients with IgAN. However, further research is needed to determine the precise mechanism by which this noncoding RNA is involved in IgAN.

Recent reports suggest that the overexpression of vtRNA 2-1 contributes to a reduction in intercellular junctions, leading to the disruption of the intestinal epithelial barrier in patients with inflammatory bowel diseases. Notably, vtRNA 2‐1 interacts with human antigen R (HuR), an RNA-binding protein (RBP) that enhances mRNA stability and the translation of intercellular junction proteins, such as claudin 1 and occludin. Ectopic expression of vtRNA 2-1 decreases the interaction between HuR and the mRNAs of intercellular junction proteins, thereby suppressing cell proliferation in primary cultures of intestinal organoids and inhibiting intestinal mucosal growth in mouse models. These effects contribute to epithelial barrier dysfunction and sepsis-related stress [103]. The HuR protein has been strongly suggested to be a mediator of the inflammatory response. In macrophages, HuR can interact with several inflammatory cytokines, affecting their biosynthesis at different levels. For instance, HuR can bind to TGFβ1, TNF, and Cox2 mRNA, decreasing the stability of TGFβ1 mRNA and reducing the protein levels and secretion of Cox2 and TNF during lipopolysaccharide (LPS)-induced macrophage stimulation [104]. Since the interaction between HuR and vtRNA 2-1 has been reported to affect the intestinal epithelial barrier, which functions as an innate defense system against pathogens, further exploration of the effect of this interaction on the expression of inflammatory cytokines is needed.

Vault particles can affect the expression of factors in innate immune cells, especially in macrophages and dendritic cells, and these cells express the highest levels of these proteins [105–107]. MVP function has been linked to the TNFα-mediated maturation of monocytes into dendritic cells (DCs). It has been reported that the overexpression of MVP plays a role in this process. Inhibiting these MVPs not only reduces the expression of differentiation markers on dendritic cells but also decreases their ability to induce antigen-specific T-cell proliferation and interferon-γ release. This finding suggests MVP is associated with dendritic cell maturation and, consequently, immune responses [106]. During dendritic cell development in murine models, an increase in MVP expression is observed. However, knockout of MVP in mice did not change dendritic cell migration or antigen presentation, suggesting that MVP could have other roles in the development of dendritic cells [108]. However, mice lacking MVP are more susceptible to infections by the bacterium *Pseudomonas aeruginosa*. This susceptibility is attributed to the inability of these mice to activate the NF-kB signaling pathway, secrete IL-8, and undergo cell apoptosis [109]. These findings highlight the importance of vault particles in innate immunity.

## Vault RNAS and viral infections

The expression of vtRNAs has been implicated in viral infections caused by DNA and RNA viruses [46, 81, 87, 110–112]. Table 1 shows a summary. The role of vtRNAs during viral infections has been examined in Epstein–Barr virus (EBV)-infected B cells, and vtRNA 1-1 was more upregulated than miRNAs, as no comparable changes were observed in response to human immunodeficiency virus (HIV) infection [110]. This difference is likely attributed to the specific mechanism by which each virus hijacks antiviral molecules [113]. In situ hybridization using antisense probes against vtRNA 1-1 showed localization in proximity to the nucleus in EBV-infected B cells, indicating a role in nucleocytoplasmic transport during infection [110].

**Table 1:** Expression and effects of vtRNAs during viral infections

	**vtRNA**	**vtRNA expression**	**Virus**	**Model**	**Effect**	**References**
1	vtRNA 2-1	Upregulated	± HSV-1	A549 cell line	NR	[112]
		Upregulated	‡SeV	A549 cell line	NR	[112]
2	vtRNA 1-1	Upregulated	± EBV	Burkitt lymphoma cell line	Pro-viral activity by activating the transcription factor NF-κB and increasing antiapoptotic proteins	[87, 110]
		Upregulated	± HSV-1	A549 cell line	NR	[112]
		Upregulated	± KSHV	BC-3 cell line	5ʹ-PPP-vtRNAs exhibit anti-viral activity by enhancing the RIG-I response	[81]
		Upregulated	‡IAV	A549 cell line	Pro-viral activity by inhibiting PKR activation	[112]
		Upregulated	‡SeV	A549 cell line	NR	[112]
3	vtRNA 1-2	Upregulated	± EBV	Burkitt lymphoma cell line	NR	[110]
		Upregulated	± HSV-1	A549 cell line	NR	[112]
		Upregulated	± KSHV	BC-3 cell line	5ʹ-PPP-vtRNAs exhibit anti-viral activity by enhancing the RIG-I response	[81]
		Upregulated	‡ IAV	A549 cell line	Pro-viral activity by inhibiting PKR activation	[112]
		Upregulated	‡SeV	A549 cell line	NR	[112]
4	vtRNA 1-3	Upregulated	± EBV	Burkitt lymphoma cell line	NR	[110]
		Upregulated	± HSV-1	A549 cell line	NR	[112]
		Upregulated	± KSHV	BC-3 cell line	5ʹ-PPP-vtRNAs exhibit anti-viral activity by enhancing the RIG-I response	[81]
		Upregulated	‡IAV	A549 cell line	Pro-viral activity by inhibiting PKR activation	[112]
		Upregulated	‡SeV	A549 cell line	NR	[112]

± DNA viral genome

^‡^RNA viral genome

NR = Not reported

Additionally, it has been suggested that the 5ʹ-PPP ends of vtRNAs play crucial roles in RNA sensor recognition [47]. This recognition can activate the antiviral response against a broad spectrum of viruses. For example, during the active lytic phase of Kaposi's sarcoma-associated herpesvirus (KSHV) infection in the B-lymphocyte line BC-3, 5ʹ-PPP-vtRNA accumulates due to reduced DUSP11, leading to activation of the antiviral response. In contrast, during the latency phase of KSHV infection, DUSP11 is expressed, resulting in the conversion of vtRNAs to 5ʹ-P, which is not recognized by RIG-I, thereby decreasing its immunogenic activity [81]. A similar effect of DUSP11 on RNA viruses such as vesicular stomatitis virus (VSV) and Sindbis virus (SINV) has been observed in the A549 cell line. This effect was confirmed in mouse models lacking DUSP11, which exhibited lower VSV viral titers due to increased expression of 5ʹ-PPP-RNAs and increased interferon response activity [46].

In contrast, vtRNAs can exert a pro-viral effect during viral replication. For instance, a reduction in the expression of vtRNA 1-1, 1-2, and 1-3 in A549 cells infected with influenza A virus (IAV) leads to a decrease in viral replication. This effect is consistent across various viruses, including Sendai virus (SeV) and Herpes simplex virus 1 (HSV-1), suggesting that these viruses have pro-viral effects. Additionally, the upregulation of vtRNA 1-1, 1-2, and 1-3 is facilitated by the NS1 IAV protein, resulting in the inhibition of the double-stranded (ds) RNA-dependent protein kinase PKR. Curiously, MVP levels are reduced during IAV infection in the same cell line; however, the role of MVP in this process is unclear [112]. Given that vtRNAs bind 5% of MVP during EBV infection [111], this decrease may occur because vtRNA molecules are mostly exert pro-viral effects in their free form.

Moreover, in the Burkitt lymphoma cell line, latent membrane protein 1 (LMP1) of EBV can induce the expression of vtRNA 1-1 by activating the transcription factor NF-κB and increasing the expression of antiapoptotic proteins, such as Bcl-xL (apoptotic protein with CARD (ARC) and B-cell lymphoma-extra-large), and promote the inflammatory response mediated by TNF/TNFR superfamily members [87]. Apoptosis, which is essential for development and tissue homeostasis, is also crucial for restricting viral infections. As a viral defense mechanism in multicellular organisms, programmed death disrupts tissues and prevents the spread of viruses [114]. Because vtRNA 1-1 activates antiapoptotic proteins, this noncoding RNA may be involved in NF-κB-mediated cell proliferation, contributing to the establishment of EBV infection.

## Vault RNAS in other cellular processes

vtRNAs are involved in diverse cellular processes, including innate immunity [84, 97, 98, 103], cell differentiation [115], cell survival [116], and cellular homeostasis [87, 117]. The expression of vtRNAs has been associated with specific tissues and cells depending on their physiological state. These noncoding RNAs can be expressed by many cells [105, 118]. See Table 2 for a summary. Typically, vtRNAs are associated with cellular processes other than immunity. This section aims to explore additional cellular events associated with vtRNAs and establish potential connections between these functions and the immune system.

**Table 2:** Summary of functional roles of vtRNAs in cellular processes

	**vtRNA**	**Related process**	**Model**	**Effect**	**References**
1	vtRNA 2-1	Cell differentiation	Sh-SY5Y cells	Targets neurodevelopment-related pathways through svtRNA2-1a	[125]
		Cell proliferation	Primary culture of CD4^+^ T cells	Inhibits PKR phosphorylation, suppressing CD4+ T-cell proliferation by reducing TGF-β expression	[98]
		Cell proliferation	MMNK1 cells, and CCA cells	Inhibits PKR phosphorylation, suppressing cellular proliferation by deactivating the NF-κB pathway	[84]
		Cell proliferation	HMEC cells	Enhances transcriptional levels of MYC oncogene	[43]
		Innate immunity	Human keratinocytes	Inhibits PKR phosphorylation, decreasing proinflammatory cytokines TNF-α, MMP-9, and COX-2 expression	[97]
		Innate immunity	Intestinal organoids and mouse models	Interacts with HuR, disrupting the remodeling of the intestinal epithelial barrier	[103]
2	vtRNA 1-1	Apoptosis	Burkitt lymphoma cell line	Enhances resistance to apoptosis by modulating NF-κB translocation and stimulating the transcription of anti-apoptotic markers	[87]
		Apoptosis	HeLa cells	Activates the PI3K/Akt and MAPK/ERK pro-survival signaling pathways	[127]
		Autophagy	HuH-7 cells	Binds directly to p62, preventing the activation of autophagy	[117, 143, 148]
		Cell differentiation	Cortical neurons	Activates the MAPK pathway for the establishment of axon/dendrite polarity	[120]
		Cell differentiation	HEK293 cells	svtRNA-4 targets CACNG7 and CACNG8 mRNAs associated with neurodevelopmental disorders	[73]
		Cell proliferation	CCK-8 Cells	Binds to PSF establishing a proliferative and drug-resistant phenotype	[129]
		Innate immunity	Burkitt lymphoma cell line	Stimulates proinflammatory cytokines mediated by members of the TNFR superfamily	[87]
3	vtRNA 1-2	Cell proliferation	HEK293T cells	svtRNA 1-2 targets pathways related to cell adhesion and cell membrane physiology, influencing the proliferation phenotype	[65]

### Cell differentiation

In the context of cell differentiation, vault RNAs expression is increased, especially in embryonic stem cells [41]. In particular, vtRNAs have been associated with nervous system development. Previous reports have demonstrated that the expression of vtRNA 1-1 enhances activation of the mitogen-activated protein kinase (MAPK) pathway in cultured cortical neurons [119]. The MAPK pathway can activate extracellular signal-regulated kinases 1 and 2 (ERK1 and ERK2), thereby leading to the regulation of local protein synthesis and the formation of axon/dendrite polarity [120]. MAPK plays a crucial role in multiple facets of the immune response, including innate immune activation, immune cell proliferation, and the orchestrated regulation of cell death in response to the completion of immune functions [121]. For example, MAPK mediates the expression of the cytokines TNFα, IL-6, IL-1α, and IL-1α, which are important for macrophage activity and are essential for activating and differentiating CD4^+^ and CD8^+^ T cells [121–124]. Given that vtRNA 1-1 can upregulate the MAPK pathway, these findings strongly suggest the participation of this noncoding RNA in the immune response.

It has been proposed that small fragments derived from vtRNAs are involved in neurodevelopment. Notably, svtRNA-4 from vtRNA 1-1 targets mRNAs such as CACNG7 and CACNG8 (calcium voltage-gated channel auxiliary subunit gamma 7 and 8), which are associated with neurodevelopmental disorders [73]. Additionally, vtRNA 1-2 and vtRNA 2-1 can be processed by Dicer, similar to the noncanonical processing of miRNAs. This processing contributes to the production of small vault RNAs, specifically svtRNA1-2 derived from vtRNA 1-2 and svtRNA2-1a derived from vtRNA 2-1. These small vault RNAs play roles in targeting pathways associated with nervous system development [65, 125]. The cleavage of vtRNAs involves m5C methylation mediated by the NSUN2 protein. In the absence of this protein, vtRNAs fail to undergo processing, consequently inhibiting their ability to regulate the expression of proteins involved in neurodevelopment [73, 125]. In addition, depletion of NSUN2 mediates the expression of RNA Pol III-transcribed products via ribonuclease P RNA Component H1 (RPPH1) activation, resulting in an increase in 5ʹ-PPP ncRNAs in the cytoplasm, which can be recognized by RIG-I, thereby inducing type I IFN signaling. This mechanism has been suggested to assist the cell in enhancing its interferon response and protect against RNA virus infections [126]. However, the exact mechanism by which NSUN2 and the fragmentation of vtRNA1-1 are recognized by the innate immune system have not been elucidated.

### Cell life and death

Interestingly, vtRNAs play a crucial role in cell survival mechanisms. Deletion of vtRNA 1-1 and vtRNA 1-2 increases susceptibility to apoptosis induction [65, 127], while overexpression of vtRNA-1-1 but not its paralogs vtRNA 1-2 or vtRNA 1-3 improves resistance to apoptosis by upregulating antiapoptotic markers such as nucleolar protein 3 (NOL3) and the Bcl-xL protein. These prosurvival proteins are located on the external mitochondrial membrane and are implicated in preventing intrinsic and extrinsic cell death in several cell types. In addition, when cells overexpressing vtRNA 1-1 are treated with TNF, IκB phosphorylation is increased compared to that in cells lacking vtRNA 1-1 [87]. IκB phosphorylation promotes the translocation of NF-κB into the nucleus, where it induces type I IFN and proinflammatory responses [86]; however, the activation of NF-κB has been associated with several aspects of oncogenesis, such as stimulating cancer cell proliferation, apoptosis resistance during drug treatment, and tumor metastasis [128]. These effects are similar to those reported on the expression of vtRNAs [83, 87, 117, 127, 129]

Hypermethylation of vtRNA promoter regions has been associated with decreased cancer cell proliferation [129–134]. In tumor cells, the overexpression of vtRNA 1-2 and 2-1, which is facilitated by genomic hypomethylation, results in increased basal transcription levels through RNA Pol III after binding to the MYC oncogene, which is a transcription factor that promotes cell growth and proliferation, thus positively impacting cancer prognosis [43, 118]. Moreover, the Dicer-dependent processing of small RNA fragments, particularly svRNA1-2 derived from vault RNA1-2, contributes to gene silencing by targeting protein-coding genes associated with the cell membrane and cell adhesion processes, ultimately influencing the proliferative phenotype [65].

**Fig. 3:**
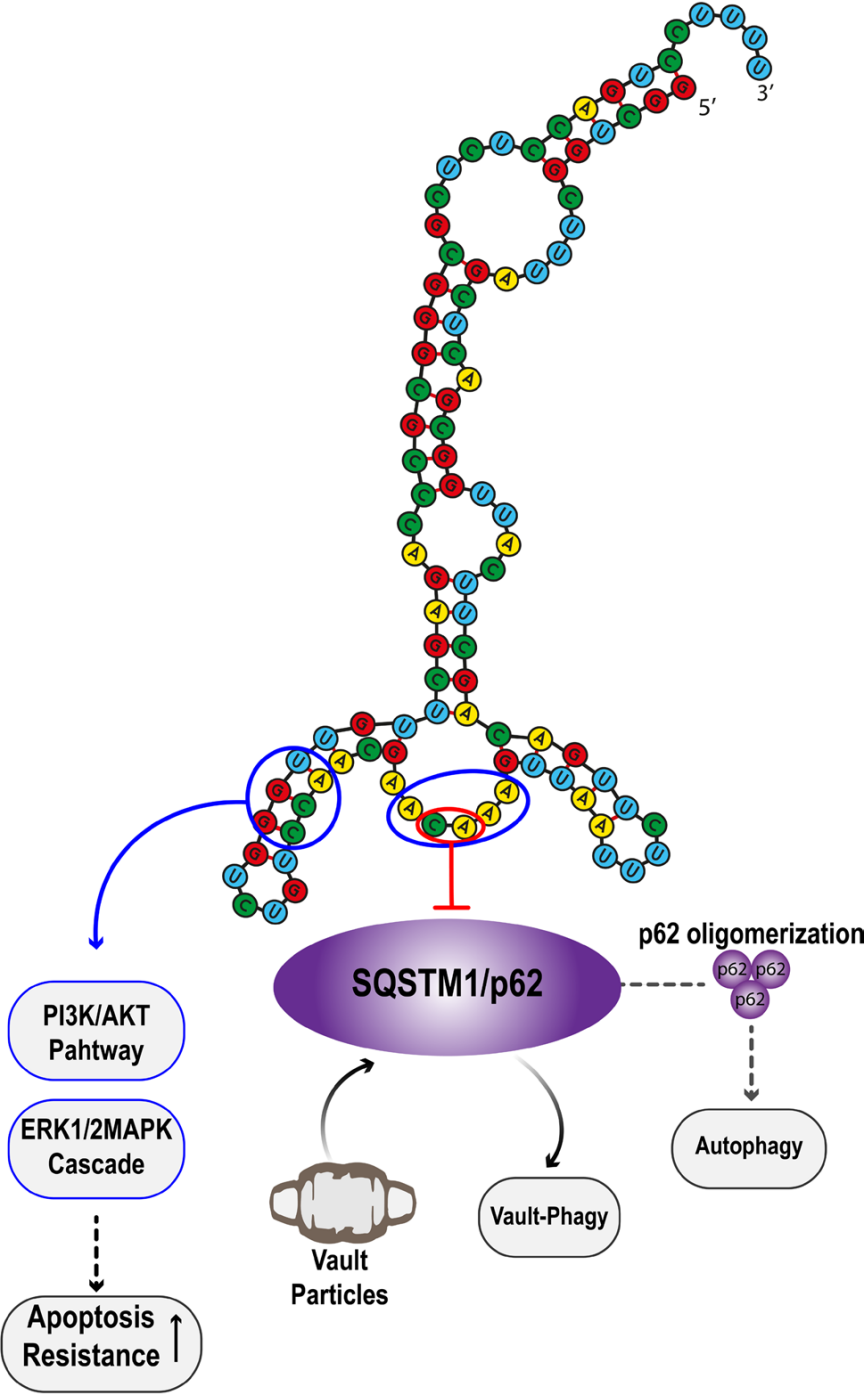
**Vault RNA 1-1 and Vault particles play a crucial role in the regulation of apoptosis and autophagy.** The stem-loop structure in the apical region of vtRNA 1-1 enhances resistance to apoptosis by modulating the PI3K/Akt and ERK1/2 MAPK signaling pathways, with critical nucleotides for apoptosis resistance encircled in blue [87, 127]. Additionally, vtRNA 1-1 inhibits autophagy by preventing p62 oligomerization, and the relevant nucleotides for this proposed role are encircled in red [138, 143]. Moreover, vault particles can interact with SQSTM1/p62, contributing to the enhancement of the vault-phagy process [141]

Notably, cell survival can be induced by vtRNA 1-1 expression through activation of the phosphatidylinositol-3-kinase (PI3K)/protein kinase B (AKT) and the ERK1/2 MAPK signaling pathways [127]. These two prosurvival signaling pathways are known to regulate various cellular processes, including apoptosis, autophagy, cell migration, adhesion, and cellular immunity [135–137]. Interestingly, an analysis of the vtRNA 1-1 structure demonstrated a secondary structure featuring a single-stranded loop region and a stem loop from the apical region of this RNA (Fig. 3). Mutational assays targeting this RNA segment have shown an increase in apoptosis in HeLa cells treated with the drug chloroquine and subjected to nutrient deprivation using starvation medium [128]. Moreover, this domain has been shown to interact with p62, a protein involved in autophagy [138]. As a result, it may also serve as a central point for interactions with other RBPs (Fig. 3). This binding site could play a crucial role in immune regulation, as it is recognized by MAPK proteins involved in diverse immune processes [137]. The presence of the PI3K protein is equally vital for immune function. For instance, this protein is essential for controlling lymphocyte differentiation, signaling, and chemotaxis. It also activates Toll-like receptors (TLRs), interleukin (IL)-1 receptors, and members of the tumor necrosis factor receptor (TNFR) family in macrophages and dendritic cells (DCs) [139]. All of these observations strongly suggest the association of vtRNA 1-1 with immunity.

### Autophagy and lysosomal regulation

vtRNAs maintain cellular physiological homeostasis by modulating autophagy and lysosomal activity. This function is exemplified in cells overexpressing vtRNA 1-1, which directly binds to the p62 receptor, which is also known as the sequestosome 1 protein SQSTM1/p62 (Fig. 3) [138]. SQSTM1/p62 is a cargo protein that is responsible for maintaining proteostasis by delivering protein targets to the phagosome [140]. The interaction between vtRNA 1-1 and SQSTM1/p62 effectively prevents protein oligomerization and the activation of autophagy, even under physiological stress such as starvation [117, 138]. Interestingly, it has been reported that MVP can directly bind to SQSTM1/p62, allowing its degradation by selective autophagy. This process is called “vault-phagy”, and mouse models lacking this process develop nonalcoholic steatohepatitis (NASH)-derived hepatocellular carcinoma [141].

Autophagy and lysosomal function have been evolutionarily conserved. During autophagy, various cytoplasmic components, including cytosolic proteins and organelles, are encapsulated within autophagosomes. Simultaneously, a cytosolic form of microtubule-associated protein 1A/1B-light chain 3 (LC3-I) conjugates with phosphatidylethanolamine, giving rise to the LC3-phosphatidylethanolamine conjugate (LC3-II), which is incorporated into autophagosomal membranes. Autophagosomes subsequently merge with lysosomes to form autolysosomes, and lysosomal hydrolases degrade the contents of autophagosomes. Moreover, LC3-II within the autolysosomal lumen undergoes degradation [140, 142]. The loss of vtRNA 1-1 results in the accumulation of LC3-II, indicating impaired autophagosomal formation. In addition, there were reductions in the network genes transcription factor EB (TFEB) and the CLEAR (Coordinated Lysosomal Expression and Regulation), which are crucial for lysosomal biogenesis. Consequently, lysosomal pH becomes more alkaline, leading to dysfunction in lysosomal compartments [143].

Autophagy is a crucial component of the innate immune system. Activation of TLRs promotes pathogen clearance through the induction of autophagy. In RAW 264.7 macrophages, stimulation with various Toll-like receptor (TLR) ligands induces autophagy, as evidenced by the increased levels of LC3-II after stimulation [144]. TLR 7 has been suggested to participate in the recognition of viral ssRNA [150], as well as in the recognition of endogenous RNAs, such as U11 snRNA [146] and fragments derived from YRNAs (s-RNYs) [147]. U11snRNA robustly activates the TLR7 pathway and induces arthritis in mouse models [146]. In cultured monocytes/macrophages, s-RNYs activate the NF-κB signaling pathway and caspase 3-dependent cell death pathways, ultimately promoting inflammatory responses and cell death [147]. Considering that vtRNAs activate autophagy and the NF-κB signaling pathway, it is plausible that these receptors also participate in the recognition of these noncoding RNAs. However, future experiments are required to examine this role.

## Therapeutic applications of vault RNAS

Vault RNAs have been correlated with several human diseases, including neurodegenerative diseases, cancer, nephropathies, autoimmune diseases, and metabolic disorders. Due to these associations, these noncoding RNAs have been proposed to be potential therapeutic targets against these diseases [49, 50, 98, 118, 125]. For instance, the fragment svtRNA2-1a derived from vtRNA2-1 was upregulated in brain areas affected by Parkinson's disease. Additionally, mimicking svtRNA2-1a in the neuroblastoma cell line Sh-SY5Y changed the expression of several long intergenic noncoding RNAs (lincRNAs) and protein-coding genes related to neuronal differentiation and maintenance [125]. These findings suggest that regulating the expression of these factors could provide new insights into therapeutic approaches.

Interestingly, vtRNAs 1-1 and 2-1 can activate the NF-κB pathway, leading to a proliferative phenotype during cancer development [65, 79, 84, 85, 87, 98, 118, 128, 148–150] and triggering the inflammatory response [87, 97]. In the serum of patients with hematological malignancies, vtRNA 1-1 and 2-1 are predominantly expressed [151, 157]. Treatment with chemotherapeutics decreases the expression of vtRNA 1-1, suggesting a possible marker for controlling this disease [152]. Moreover, MVP has been implicated in cancer proliferation and drug resistance. The inflammatory factor IL-25 induces the expression of MVP, which activates NF-κB, reinforcing the importance of vtRNAs in innate immunity [121, 153]. These findings suggest that vtRNAs could be targets for anticancer immunity. The transcription factor NF-κB has an impact on tumor development and progression through excessive innate immune activation and abnormal cell proliferation [154, 155]. During inflammation-associated cancer, the binding of NF-κB to gene promoters induces the expression of cytokines such as TNF-α, IL-1B, and IL-6, thereby facilitating cell proliferation, invasion, angiogenesis, and macrophage polarization in several tumors [156–158]. Similarly, in ovarian tumors, TNF-α is produced in large amounts, promoting a proinflammatory tumor microenvironment and activating NF-κB signaling, which induces the expression of chemokines including CCL8, CXCL13, and CXCL20 to accelerate tumor development [159]. It has been proven that vtRNA 1-1 is a potent stimulator of NF-κB [87], and its association with cell proliferation in cancer [128] suggests that this noncoding RNA could be a strong candidate for use in anticancer immune therapies. Previous studies of HeLa cell cultures have suggested that knocking out vtRNA1-1 could result in high levels of apoptosis during prolonged starvation. Similar proliferative phenotypes were observed when using antisense oligonucleotides (ASOs) against vtRNA 1-1 [128]. This effect is likely due to the susceptibility of cells to starvation stress and inactivation of the NF-κB signaling pathway, which increase apoptosis. Given these findings, there is a broad range of potential therapies to regulate NF-κB via these noncoding RNAs.

In T-NK-cell neoplasms, Epstein–Barr virus (EBV) can persistently activate NF-κB through the viral oncoprotein LMP1, which is essential for B-cell transformation. LMP1 activates TNF receptor-associated factors (TRAFs), activating the canonical and noncanonical NF-κB pathways through IκB and RelB activation, respectively. This activation leads to the hyperactivation of TNF-α, macrophage inflammatory protein 2 (MIP-2), and interferon-inducible T-cell alpha chemoattractant (I-TAC), thereby promoting cellular transformation [160]. Interestingly, in Burkitt lymphoma-derived BL41 cells, ectopic expression of human vtRNA1-1 significantly increased resistance to apoptosis in response to stimuli such as staurosporine or topoisomerase-II inhibitors, which are potent inducers of cell death through the intrinsic or extrinsic apoptotic pathway, respectively. Conversely, when siRNAs and antisense oligonucleotides (ASO) against vtRNA 1-1 were used, the cells exhibited increased susceptibility to apoptosis following the treatments. Furthermore, it has been reported that the expression of vtRNA 1-1 is induced by the activation of NF-κB, which is mediated by the oncoprotein LMP1. Treatment of HeLa and HS578T cells with the NF-κB inhibitor IKK VII decreased the expression of vtRNA 1-1 [87]. These findings collectively suggest that vtRNA 1-1 could exert a significant impact on therapies involving the activation of inflammatory components, leading to the inhibition of tumor cell proliferation. Future improvements in our understanding of these noncoding RNAs may be valuable in developing novel RNA-based therapies.

## Concluding remarks

Comprehensively defining the role of vtRNAs in the interferon-mediated response is complex. vtRNA1-1 strongly affects the NF-κB signaling pathway, inducing proliferative phenotypes in tumor cells and promoting an inflammatory tumor microenvironment. Due to its ability to regulate cell proliferation, vtRNA 1-1 may be a therapeutic target for anticancer immune therapies. In contrast, vtRNA 2-1 can sequester the PKR protein, inhibiting activation of the NF-κB signaling pathway. Changes in the expression of vtRNA 2-1 have been implicated in diseases such as IgA nephropathy. Moreover, the interaction between OAS1 and vtRNA 2-1 is poorly understood in the context of innate immunity. Nevertheless, OAS1 is a crucial component of IFN regulation. Understanding how vtRNA 2-1 regulates OAS1 or how OAS1 regulates vtRNA 2-1 could provide valuable insights for advancements in disease control.

Finally, understanding the mechanisms of vtRNA biogenesis is important. When vtRNAs are fragmented into svtRNAs, they can regulate gene expression. These factors participate in various cellular processes, such as the development and maintenance of cell membrane physiology. However, how this process can affect innate immunity is unclear. The NSUN2 enzyme may be involved in the activation of Pol III transcripts. All the available knowledge regarding the effect of vtRNAs on innate immunity is based on the use of raw molecules. Therefore, understanding the roles of these svtRNAs in innate immunity could provide new insights into the roles of these noncoding RNAs in the inflammatory process.

## Data Availability

No data associated in the manuscript.
